# PD-1 involvement in CD8+ tumor-infiltrating lymphocytes in patients with colonic-derived peritoneal adenocarcinoma

**DOI:** 10.1590/1414-431X2025e14467

**Published:** 2025-04-14

**Authors:** Huihui Hu, Man Zhang

**Affiliations:** 1 Department of Clinical Laboratory, Beijing Shijitan Hospital, Capital Medical University, Beijing, China; 2 Beijing Key Laboratory of Urinary Cellular Molecular Diagnostics, Beijing, China; 3 Clinical Laboratory Medicine, Peking University Ninth School of Clinical Medicine, Beijing, China

**Keywords:** PD-1, CD8, Colonic-derived peritoneal adenocarcinoma, Recurrence, Flow cytometry, Multiple immunofluorescences

## Abstract

Immune checkpoint blockade with anti-programmed cell death protein 1 (PD-1) antibody has become a hot topic for the treatment of human malignancies. Here, we aimed to investigate whether the percentage of PD-1 in CD8+ tumor-infiltrating lymphocytes correlates with the progression of colonic-derived peritoneal adenocarcinoma (PA). Peripheral blood and tissue samples from 40 patients with colonic-derived PA were collected and subjected to multicolor flow cytometry analysis of the percentage of peripheral PD-1+CD8+ T cells. The multiple immunofluorescence method was used to detect the positive percentages of PD-1 and CD8 in the tissues. The enrolled patients were divided into groups by recurrence interval (less than 6 months, greater than two years) and differentiation grade (low, well/moderate). In the colonic-derived PA tissues, the percentages of cells positive for PD-1, CD8, and PD-1+CD8+ were higher in the paracancer tissues compared with cancerous tissues. PD-1+CD8+ T cells had an increased presence in peripheral blood than in tissues. Our data also indicated that colonic-derived PA patients with less than a six-month recurrence interval presented higher levels of PD-1 in CD8+ tumor-infiltrating lymphocytes in than the two-year recurrence group. The level of PD-1+CD8+T cells in the tissue correlated with the clinical outcome of colonic-derived PA. Higher percentages of PD-1+CD8+T cells correlated with a shorter progression-free survival (PFS). PD-1 in CD8+ tumor-infiltrating lymphocytes may have a good predictive value for immunotherapy of colonic-derived PA and act as the prognostic factor for PFS.

## Introduction

Colonic-derived peritoneal adenocarcinoma (PA) is the most common pathologic type of peritoneal tumor. Due to the insidious onset and relatively difficult treatment of this disease, it is crucial to find an effective clinical treatment (1). Most PAs are metastatic, and colon cancer is the most common primary lesion. The most prominent features of colonic-derived PA are diffused or localized invasive malignancy. Although clinical therapy for the colonic-derived PA in recent years has become multi-modal, most patients do not experience clinical benefits after undergoing radical resection and chemotherapy.

Immune checkpoint blockade was an innovation in cancer treatment (2-4). Although immune checkpoint therapy has achieved good remission in some cancer patients, there are few reports on its use in colonic-derived PA. Programmed cell death protein 1 (PD-1) is part of the B7/CD28 family and is mainly expressed by activated T cells. It acts as a key immune-checkpoint receptor in the regulation of immunosuppression (5,6). Immunotherapy is now considered to be the key treatment for cancer, and the PD-1 checkpoint has achieved significant progress in clinical therapy for human melanoma, lung cancer, renal cancer, and Hodgkin's lymphoma (7). Immune checkpoints expressed by T cells can negatively mediate human immunity against tumors. Lymphocyte subsets are important tools for revealing the immune status of the human body (8). More recent studies in immunotherapy have further demonstrated the synergistic effect on tumor immune response activation (9,10). PD-1 is considered as an exhaustion marker for T cells (11). Exhausted T cells express inhibitory receptors, produce fewer effector cytokines, and lose the ability to eliminate cancer (12). PD-1 also closely correlates with clinical outcomes of human cancer. Granier et al. (13) reported the existence of PD-1+ cells associated with poor prognosis in renal cell carcinoma. However, most patients have limited clinical benefits after receiving immunosuppressive therapy. The main reason is the emergence of drug resistance during treatment (14,15). For patients who develop drug resistance, receiving PD-1 immunosuppressive therapy alone is not enough (16). Our laboratory has reported that differential frequencies of PD-1 in the peripheral blood of patients with peritoneal neoplasms correlates with the different pathological types. PD-1 presents higher levels in the adenocarcinoma group compared with the mesothelioma group (17). However, there are no reports on the correlation between PD-1 and drug resistance in PA.

The purpose of the present study was to investigate whether the positive percentage of PD-1 in CD8+ tumor-infiltrating lymphocytes is associated with the progression of colonic-derived PA. We investigated the immunohistochemical result of PD-1+CD8+T cells expression in different tissue groups. Considering that malignant tumors are prone to recurrence, the focus of this article was to explore whether PD-1 in CD8+ tumor-infiltrating lymphocytes are correlated with recurrence interval and progression-free survival (PFS). We also detected the expression of circulating PD-1+CD8+T cells in the peripheral blood. Furthermore, the differential expression of PD-1+CD8+T cells in peripheral blood and tissues of colonic-derived PA was compared (18,19).

## Material and Methods

### Patients

Patients with colonic-derived PA were enrolled between December 2022 and April 2024. All the 40 patients enrolled received no previous PD-1 immune checkpoint blockade, and the pathological type was confirmed as colonic-derived PA. Patients with other tumors and immune system diseases were excluded. Peripheral blood samples (EDTA-K2 anti-coagulated) were obtained from the laboratory department, and tissue samples were collected from patients who underwent surgery at Beijing Shijitan Hospital. The enrolled patients were divided into groups by recurrence interval (less than 6 months, greater than two years) and differentiation grade (low, well/moderate). The patients' details are listed in Table 1. This study was approved by the institutional review board for the protection of human subjects, and all participants gave informed written consent.

**Table 1:** Patients' characteristics.

Parameter	n (%) or median [Q1-Q3]
Total number of patients enrolled	40
Age at diagnosis (years)	55.8 [22-82]
Gender (F/M)	15/25
Histological grade	
Low differentiation	27 (66.3%)
Moderate differentiation	7 (16.25%)
High differentiation	6 (17.50%)
Recurrence after initial surgery	
<6 months	16 (20%)
>2 years	15 (18.75%)

### Flow cytometry analysis

This experiment used the Beckman Coulter ten-color flow cytometer (Navios, USA). EDTA-coated fresh venous blood samples were collected from patients. The antibodies used were: specific antibodies against human APC CD279 (PD-1), APC/Fire™ 750 anti-human CD4, PE anti-human CD3, Pacific Blue™ anti-human CD8, and Brilliant Violet 510™ anti-human CD45 (all from Biolegend, USA). Fresh venous blood samples labeled with corresponding fluorochrome conjugated non-immune isotypes were used as negative controls. The viability of the cells was also tested by violet amine reactive dye (Invitrogen, USA). Fresh venous blood samples (200 μL) were incubated with 5 μL of each antibody. After 20 min of incubation at room temperature in the dark, blood cells were lysed with erythrolysin (Beckman Coulter) for 15 min at room temperature in the dark. After two washing steps in phosphate-buffered saline (PBS), cells were analyzed by flow cytometry. For the analyses of PD-1 on the surface of T cells, 5000 lymphocytes per tube were collected as termination condition. The analysis was performed with Kaluza software (Beckman Coulter).

### Multiple immunofluorescence stains for PD-1 in tissue sections

The paraffin-embedded tissue samples were sectioned into 5-mm slices. The sections were dewaxed with xylene for 30 min at room temperature and then dehydrated by a gradient of ethanol. After three washing steps in PBS, the tissue sections were blocked with endogenous peroxidase for 20 min at room temperature. After antigen retrieval, the samples were incubated with mouse polyclonal PD-1 antibody (Proteintech, USA) at a dilution of 1:300 at 4°C overnight. After washing steps in PBS, the sections were incubated with fluorescently labeled secondary antibodies for 40 min at room temperature, followed by three washing steps in PBS, and then 100 μL DAPI solution was added and incubated for 5 min at room temperature in the dark. After three washing steps in PBS, the tissue sections were dehydrated with a gradient of ethanol, then sealed with fluorescent tablet (Sealing medium, EURO, Germany).

Analysis was performed using TissueGnostics software (Austria). The DAPI staining kernel identifies all DAPI cores based on the strength of the threshold setting. After the analysis parameters were set, the expressions of PD-1 and CD8 in cells were analyzed using the nucleus as the starting point. The immunostaining intensity was determined by the average pixel intensity per cell relative to all pixels in a cell region of 8-bit grayscale. Using the ATHE software, a specific distance of 10 μm can be observed, and based on non-specific control samples, it can be determined that the cytoplasmic immunostaining intensity above a specific threshold is specific. The percentage of positive cells (percentage of positive cells/all nucleated cells) and the positive cell density were measured using the TissueQuest assay platform. To ensure the accuracy of the results, three independent pathologists quantified the CD8- and PD-1-positive cells.

### Statistical data analysis

The percentages of positive cells in peripheral blood and tissue sections were compared by group using independent sample *t*-test or Mann-Whitney U nonparametric test. The data are reported as medians and interquartile range. Kaplan-Meier curves were used to analyzed PFS. P<0.05 indicated a significant difference. Data were analyzed using GraphPad Prism 5.0 software (GraphPad Software, USA).

## Results

### PD-1 positive expressions in CD8+ tumor-infiltrating lymphocytes in tissue sections *vs* progression-free survival (PFS)

**Figure 1:**
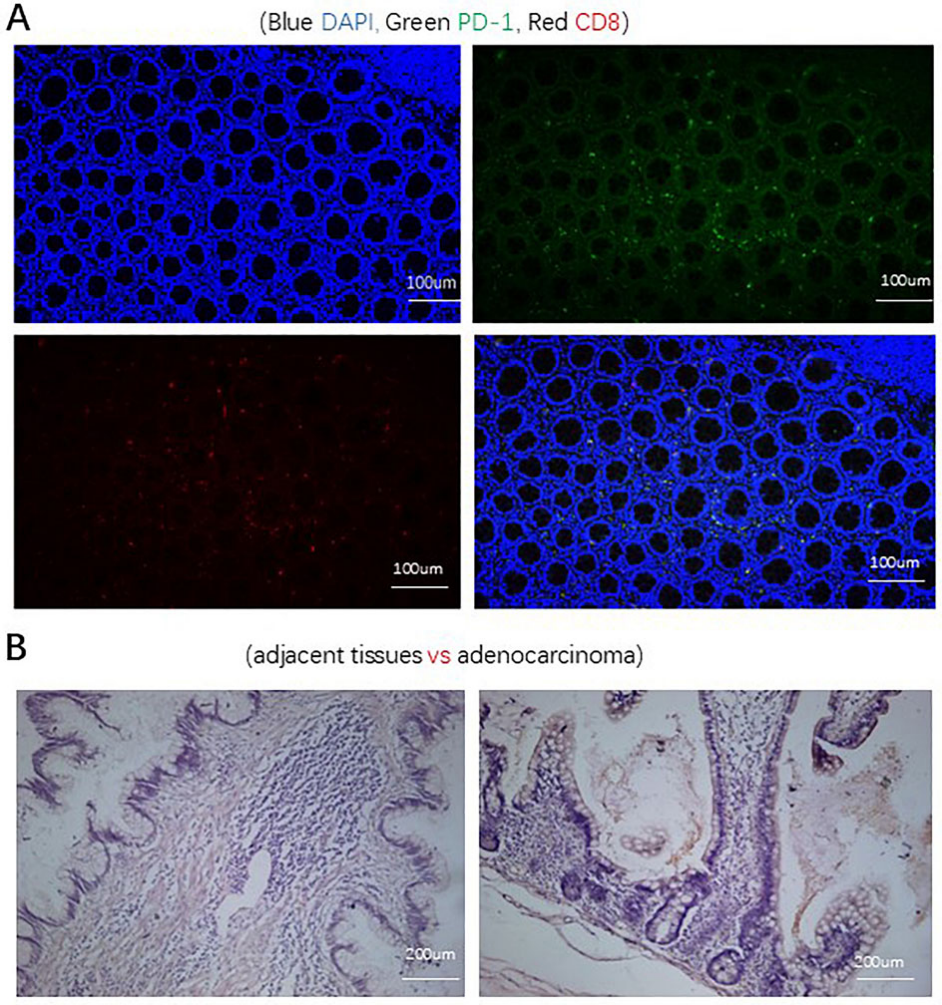
**A**, Multiple tissue fluorescence maps of PD-1 and CD8. Blue for DAPI, green for PD-1, and red for CD8. Scale bars, 100 μm. **B**, Hematoxylin eosin staining results of adjacent and adenocarcinoma tissues. Scale bars, 200 μm, ×400 magnification.

**Figure 2:**
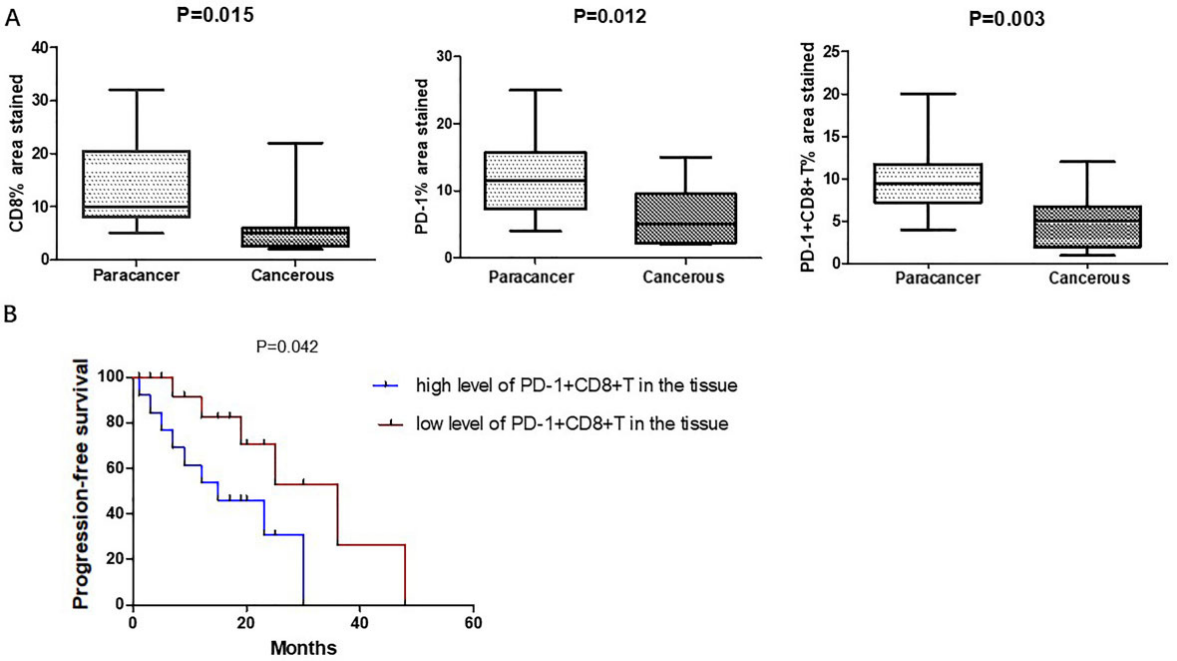
**A**, Chart of the expressions of CD8 and PD-1 of paracancer tissues and cancerous tissues. Data are reported as median and interquartile range. P<0.05 indicates statistical significance (Mann-Whitney U nonparametric test). **B**, Association between PD-1+ cells in the tissue section and progression-free survival (Kaplan-Meier curves).

The differential expression of CD8 and PD-1 in colonic-derived PA and adjacent tissues is shown in Figure 1A and B. The results showed that CD8+ (13.7 *vs* 5.9%, P=0.015), PD-1+ (12.2 *vs* 6.3%, P=0.012), and PD-1+CD8+ T cells (10.1 *vs* 4.9%, P=0.003) were higher in para-cancerous tissues than in cancerous tissues (Figure 2A).

PFS was greater amongst those with lower levels of PD-1+ (tumor-infiltrating lymphocytes (TILs) (median PFS in high level 11.7 months *vs* 24.5 months in low level, P=0.042, Figure 2B). The level of PD-1+ in the tissue sections correlated with PFS.

### Circulating PD-1+CD8+T cells in peripheral blood

**Figure 3:**
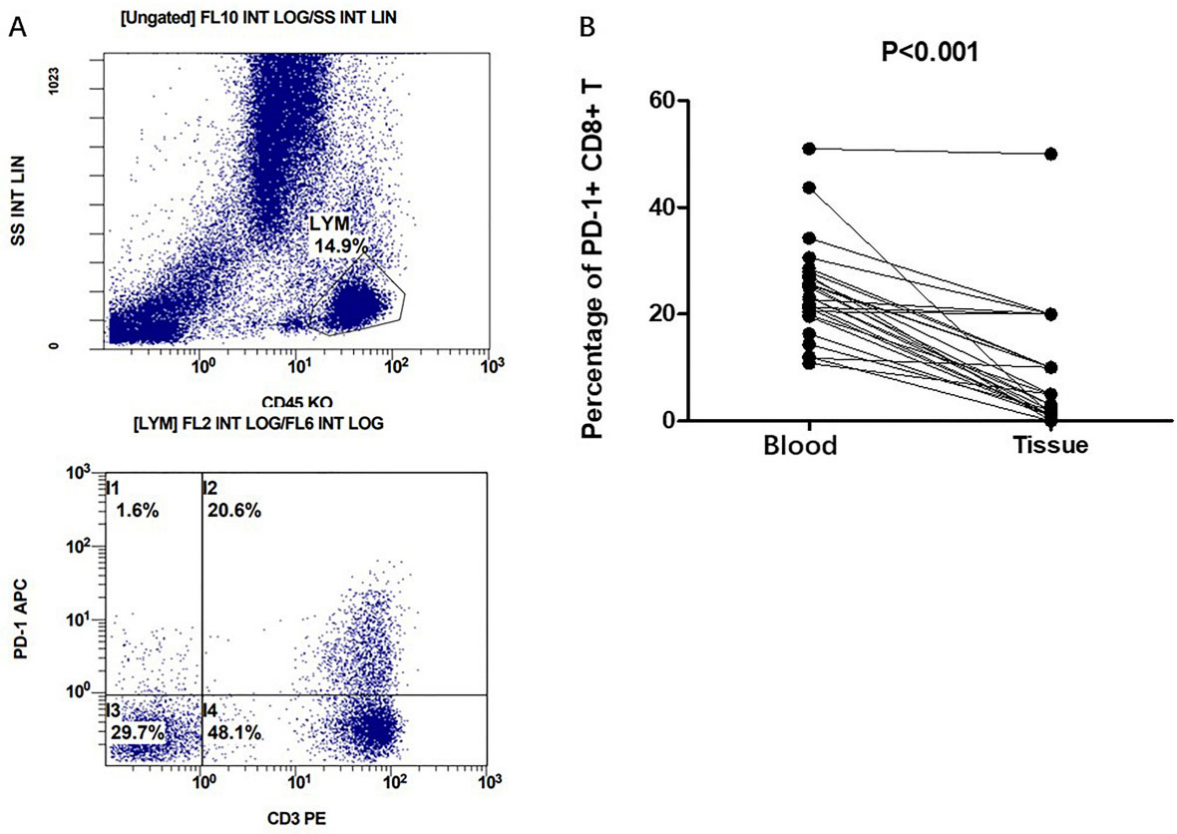
Circulating PD-1+CD8+ T cells in peripheral blood by flow cytometry. **A**, Flow diagram of PD-1+CD8+ T cells. **B**, Differential percentages of PD-1+CD8+ T cells in peripheral blood and tissue sections of the same patient (Student’s *t*-test).

The frequency of circulating PD-1+CD8+T cells in peripheral blood was explored by flow cytometry (Figure 3A). We compared the differential percentages of PD-1+CD8+ T cells in the peripheral blood and tissue sections of the same patient. PD-1+CD8+ T cells were lower in TILs than circulating lymphocytes in peripheral blood (24.3 *vs* 8.9%, P<0.001, Figure 3B).

### The percentage of PD-1+CD8+ T cells in tissue sections correlated with differentiation and recurrence interval

**Figure 4:**
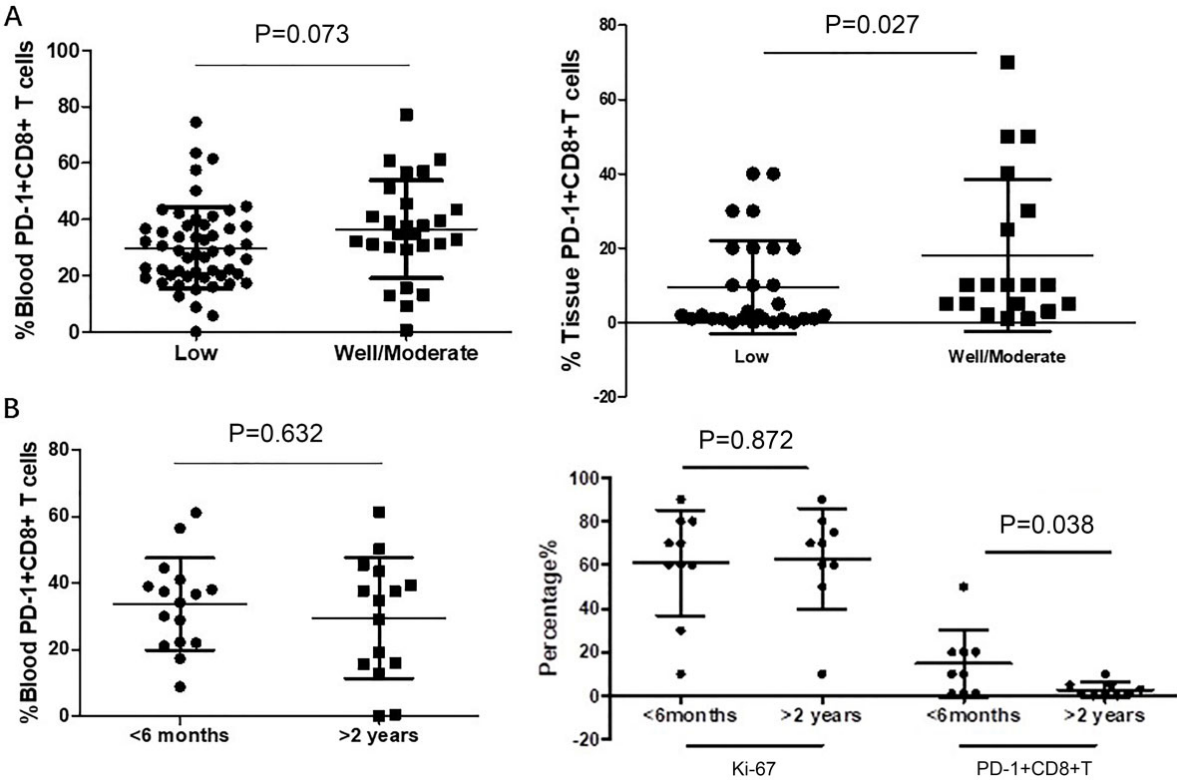
Differential percentages of PD-1+CD8+ T cells in tissue sections correlated with differentiation grade and recurrence interval of colonic-derived peritoneal adenocarcinoma patients. **A**, Differentiation grade of PD-1+CD8+ T cells in peripheral blood and tissue sections. **B**, Recurrence interval of PD-1+CD8+ T cells in peripheral blood and tissue sections. Horizontal lines represent median and interquartile range (Mann-Whitney U test).

The association of PD-1+CD8+ T cells in peripheral blood and in tissue sections with differentiation grade (low, moderate/well) were investigated. Compared with low grade, the patients with well/moderate grade adenocarcinoma had increased expression of PD-1+ CD8+ T lymphocytes (10.6 *vs* 16.1%, P=0.027, Figure 4A). No association was found with PD-1+CD8+ T cells in peripheral blood (Figure 4A).

The recurrence interval after the first surgery was divided into two groups (less than 6 months and greater than two years). The percentage of Ki-67-positive cells had no statistical difference between the above groups (61.00 *vs* 62.78%; P=0.872, Figure 4B). The percentage of PD-1+CD8+ T cells in tissues was higher in recurrence in the less than 6 months group than that in the group of recurrence in more than 2 years (11.27 *vs* 7.25%; P=0.038, Figure 4B). PD-1+CD8+T cells in the peripheral blood had no relationship with the recurrence interval (Figure 4B).

## Discussion

Immune checkpoint blockade with anti-PD-1 has become a hot topic for the treatment of human malignancies (20,21). As its name implies, PD-1 is encoded by the *PDCD1* gene and was initially identified to induce programmed cell death (22). The application of immune checkpoint blockade with anti-PD-1 in colonic-derived peritoneal neoplasms is rarely reported, and it remains unclear how PD-1 impacts the prognosis of colonic-derived PA. Antibodies that block the PD-1 pathway have demonstrated antitumor activity in cancer patients and have gained approval by the Food and Drug Administration (USA) for treatment of several different cancers (23,24). Kamphorst et al. (25) showed that 70% of lung cancer patients with disease progression had a delayed PD-1+CD8+ T cell response, whereas 80% of patients with clinical benefit expressed PD-1+CD8+ T cell. Furthermore, it has been reported by Bekos et al. (26) that, in ovarian tumor tissues, the percentages of CD8-, PD-1-, and PD-L1-expressing subpopulations of TILs differed between primary tumor tissues and metastatic intraperitoneal metastases. Moreover, in peripheral blood of patients with acute-on-chronic liver failure, PD-1 induced T lymphocyte dysfunction, which might involve glycolysis inhibition. Yang et al. (27) showed that PD-1 and Tim-3 were co-expressed and their crosstalk regulated T cell exhaustion and suggested galectin-9 as a promising target for immunotherapy.

Immune escape plays an important role in the occurrence and development of tumors (28). In gastric or gastroesophageal junction adenocarcinoma, the use of PD-1/PD-L1 inhibitors is gradually shifting from third-line to first-line treatment (29). The clinical response rate of a PD-1 inhibitor combined with chemotherapy in the treatment of gastrointestinal PA can be increased to 50 to 60% (30,31). Programmed cell death-ligand 1 (PD-L1) is the main ligand of PD-1, it is upregulated in various solid tumors, and it plays a pivotal role in inhibiting cytokine production and the cytolytic activity of PD-1+ tumor-infiltrating CD8+ T cells (32-34). Several studies have reported the role of PD-1/PD-L1 in the tumor microenvironment and their association with poorer prognosis in gastric and colorectal cancers (35,36). Hoshimoto et al. (37) have demonstrated that immune checkpoint inhibitors may improve the prognosis of small bowel adenocarcinoma patients with low FoxP3/CD8 ratio and PD-L2 expression.

Our data strengthened the assumption that PD-1 in CD8+ tumor-infiltrating lymphocytes may have a good predictive value for immunotherapy of colonic-derived PA and act as a prognostic factor for PFS. Compared with the positive percentage of PD-1 in peripheral blood, PD-1 positive expression in tissues was lower. The data suggested a mechanism of PD-1 involving immune tolerance at a tissue level in colonic-derived PA. Arai et al. (38) reported that the increased percentage of PD-1 in circulating CD4+ and CD8+ T cells was associated with dysfunction of cell immunity after colorectal cancer surgery. In epithelial ovarian cancer patients, the infiltration rates of PD-1 and PD-L1 were different between primary ovarian tumor tissue and metastatic intraperitoneal implants (26). Our results suggested that the percentage of PD-1+CD8+ T cells at the tissue level was closely linked with the recurrence interval of colonic-derived PA.

Since Ki-67 index was correlated with tumor malignancy (39,40), we assessed the correlation between PD-1+CD8+ T cells, Ki-67, and the progression of colonic-derived PA. Compared with Ki-67, PD-1 in CD8+ TILs was more correlated with colonic-derived PA. Drug resistance is a major obstacle to successful chemotherapy, and the recurrent tumor is always accompanied by drug tolerance. There are many studies about the monitoring and avoidance of drug resistance in human cancer. However, drug resistance seems to be a multigene phenomenon, but the exact mechanism is unclear. PD-1 was involved in a network of drug resistance-related signaling pathways and played an indispensable role in drug resistance formation. These results were consistent with the correlation between PD-1 and PFS.

These findings suggested that PD-1 in the CD8+ TILs in tissue sections could be a predictive biomarker for colonic-derived PA progression. Nonetheless, the present study had several limitations. Firstly, additional research using a larger sample size is necessary to validate these results. Secondly, the exact mechanism of PD-1 in the occurrence, development, and recurrence of colonic-derived PA will be studied further. Further studies on the molecular mechanism of PD-1-mediated immunosuppression may provide valuable guidance to the novel treatment for the clinical therapy of colonic-derived PA. Thirdly, more methods such as fluorescent quantitative PCR are needed to investigate the prognostic role of PD-1 in colonic-derived PA.

## Conclusion

We demonstrated that PD-1 in CD8+ TILs was associated with the differentiation grade and recurrence interval of colonic-derived PA. These results indicated that PD-1 in CD8+ TILs may have a good predictive value for the immunotherapy of colonic-derived PA, and act as a prognostic factor for PFS.
